# Rotator Cuff Repair With “BicepBrace” Biceps Tendon Augmentation

**DOI:** 10.1016/j.eats.2024.103395

**Published:** 2024-12-26

**Authors:** Theodore Joaquin, Gregory Perraut, Brandon Crowley, Evan Argintar

**Affiliations:** a Georgetown University School of Medicine, Washington, DC, U.S.A.; b Department of Orthopedic Surgery, MedStar Washington Hospital Center, Washington, DC, U.S.A.

## Abstract

Massive rotator cuff tears comprise a large portion of rotator cuff injuries, yet their surgical repair is highly prone to failure. In this Technical Note, a biceps tendon transfer technique is described for the repair of massive rotator cuff tears. This method of repair is an easy, cheap, accessible technique created to improve clinical outcomes in patients with massive tears. More research, both clinical and biomechanical, is needed to evaluate the utility of this augmentation with rotator cuff repair of all sizes.

Rotator cuff tears are prevalent orthopaedic injuries that affect approximately 20% of individuals at some point in their lives.^1^, ^2^, ^3^ The most serious iteration of these injuries is the massive rotator cuff tear. Massive rotator cuff tears account for 40% of all rotator cuff injuries.^4^ A comprehensive review by Di Benedetto et al.^5^ in 2021 detailed the techniques available for repairing these extensive tears. These techniques include debridement, partial repair, superior capsular reconstruction (SCR), tendon transfer, tuberoplasty, and some combination of these options. Although massive rotator cuff tears represent a minority of cuff injuries, their repair surgeries are far more likely to result in failure and require revision.

In this Technical Note, we outline our biceps tendon transfer technique, which can be used either as an alternative to, or to augment, rotator cuff repair. This technique is unique in that it allows for simultaneous proximal biceps tendon autograft SCR, distal biceps tenodesis, and tuberoplasty, if desired.

Using the long head of the biceps tendon to enhance the stability of rotator cuff repairs is not a new concept (Table 1).^6^, ^7^, ^8^, ^9^, ^10^, ^11^, ^12^, ^13^, ^14^, ^15^, ^16^, ^17^, ^18^, ^19^, ^20^, ^21^, ^22^, ^23^, ^24^, ^25^, ^26^, ^27^, ^28^, ^29^, ^30^, ^31^, ^32^, ^33^, ^34^, ^35^, ^36^, ^37^ However, our approach involves fixating the medial aspect of the tenotomized biceps tendon to at least 2 points of fixation on the greater tuberosity. In addition, the tenodesis of the lateral biceps tendon prevents the comorbidities and deformity typically observed after biceps tenotomy. Lastly, if desired, tuberoplasty may be performed laterally on the greater tuberosity.

**Table 1:** Alternative Uses of the Long Head of the Biceps Tendon in Arthroscopic Repairs of Massively Torn Rotator Cuffs

Uses	Number of Publications	References
Cuff augmentation incorporating biceps tendon to reduce strain	15	6, 7, 8, 9, 10, 11, 12, 13, 14, 15, 16, 17, 18, 19, 20
Modified superior capsular reconstruction	9	21, 22, 23, 24, 25, 26, 27, 28, 29
Reinforcement of repair site with patch or graft	4	30, 31, 32, 33
Modified anterior cable Reconstruction	4	34, 35, 36, 37

### Indications

Although there are several competing definitions,^38^, ^39^, ^40^ this study considers tears to be massive when they involve 2 or more of the rotator cuff tendons, with retraction to the level of the glenoid. All of these tears are nonrepairable because of a combination of size, tissue quality, retraction, muscle atrophy, and fatty infiltration. This technique can be used with partial and full rotator cuff repair.

### Surgical Technique

We describe a detailed technique of the procedure in Video 1. The technique described is similar to that of Kim et al.^41^ in their biceps rerouting article. In contrast, our technique uses tenotomy, tendon transfer, and tenodesis combined with a double-row repair.

### Approach

Patients are positioned in a lateral beach-chair position with their arm in traction (Fig 1). The upper extremity is prepped and draped using standard technique. An initial posterolateral portal is created. Under arthroscopic guidance, an anterosuperior portal is created.

**Fig 1:**
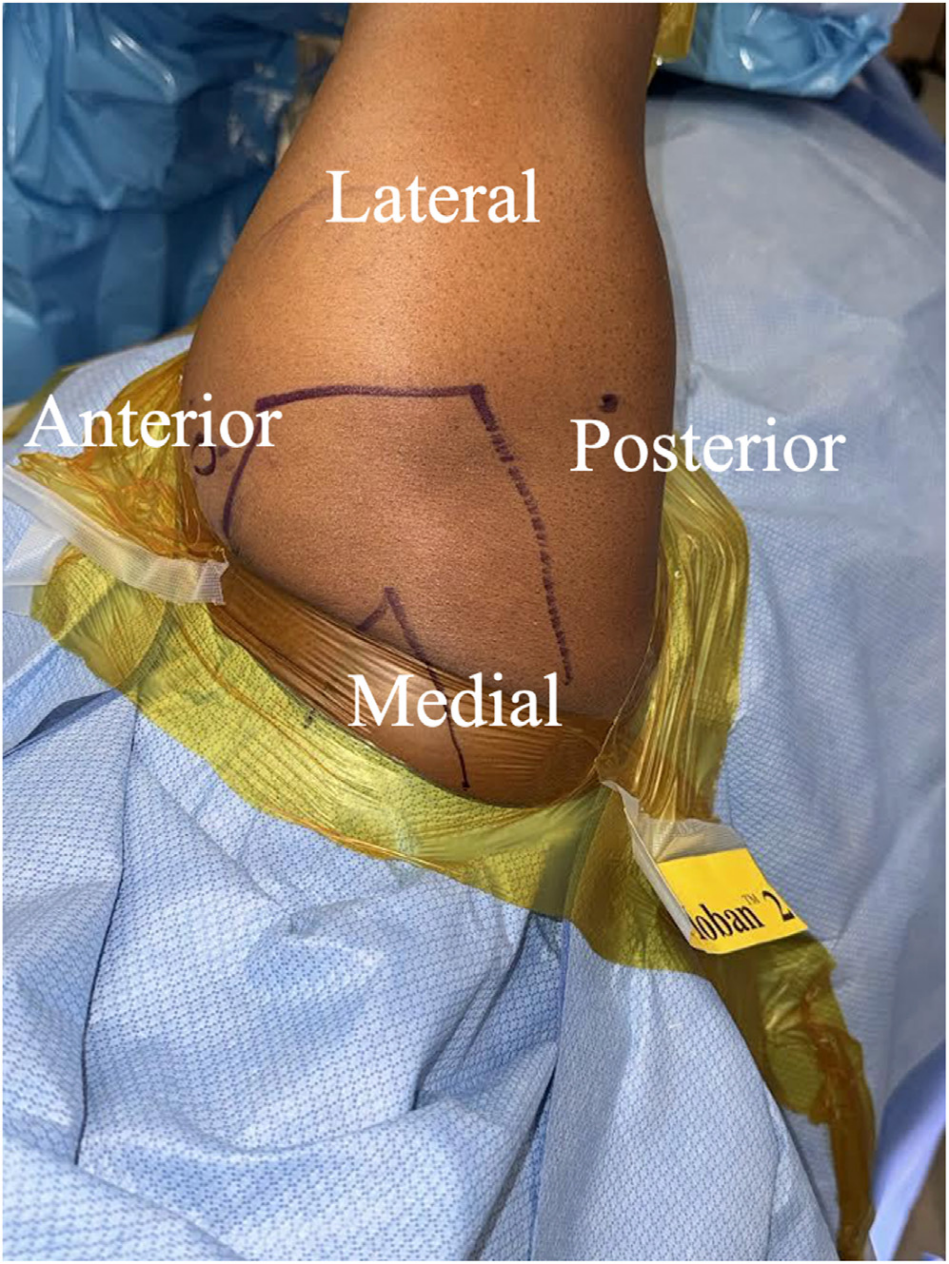
Superior view of a patient's right shoulder prepared for surgery. The patient is seated in the lateral beach-chair position. Preoperative planning includes marking the bony structures of the shoulder.

### Tendon Preparation

Initially, using a self-grasping suture passer, a suture is placed through the biceps tendon at the level where it traverses the humeral head approximately 2 to 3 cm lateral to its insertion on the glenoid (Fig 2). The center of the suture is passed through the tendon and the 2 tails are then pulled through the loop to form a luggage handle tag, allowing the physician to use the suture for traction (Fig 3). A percutaneous incision is made through the Neviaser portal, just lateral to the medial aspect of the acromion, and the biceps tendon suture is extracted through this portal. Tension is placed on the suture, pulling the biceps tendon intra-articular, exposing more tendon length (Fig 4). Using the self-grasping suture passer, a second suture is placed through the lateral portion of the exposed biceps tendon and similarly secured using the luggage-handle technique, as described previously (Fig 5). If tuberoplasty is desired, subacromial debridement of the biceps tendon sheath can be performed to maximize the length of usable distal biceps tendon.

**Fig 2:**
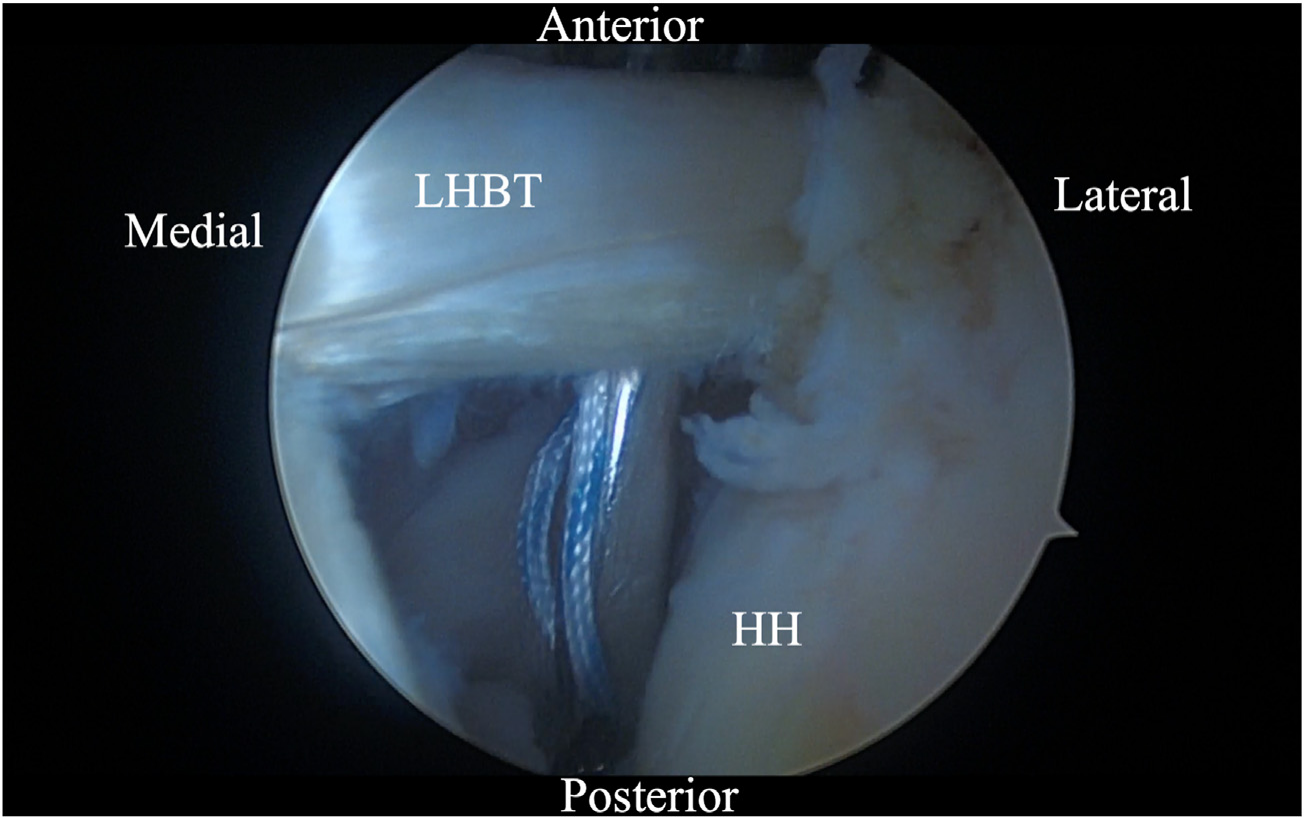
Arthroscopic image of right shoulder (lateral beach-chair position) viewed through the subacromial posterior portal showing a self-grasping suture passer placing a suture through the LHBT at the level where it traverses the humeral head approximately 2 to 3 cm lateral to its insertion on the glenoid. (HH, humeral head; LHBT, long head of the biceps tendon.)

**Fig 3:**
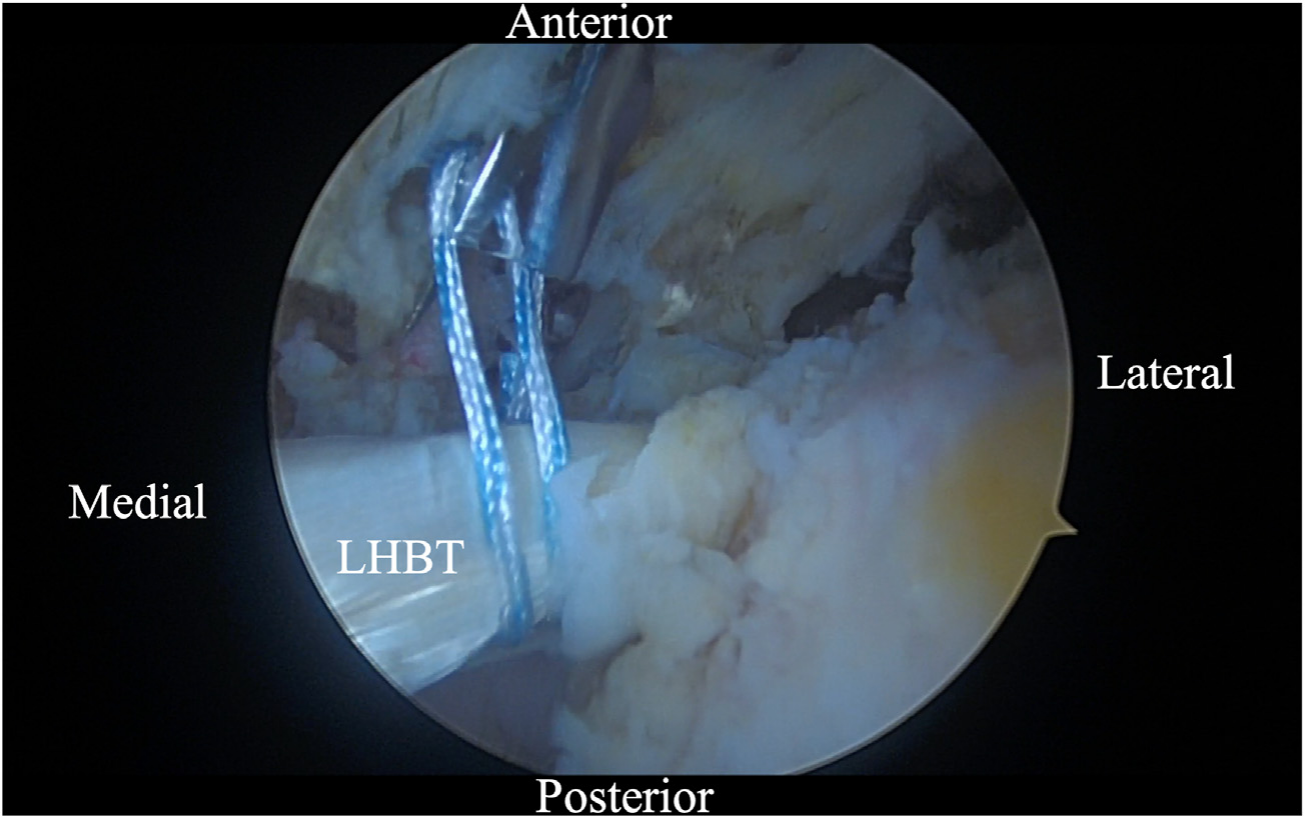
Arthroscopic image of right shoulder (lateral beach-chair position) viewed through the subacromial posterior portal showing the 2 tails of suture being pulled through the loop of the suture passer to form a luggage-handle tag on the LHBT. (LHBT, long head of the biceps tendon.)

**Fig 4:**
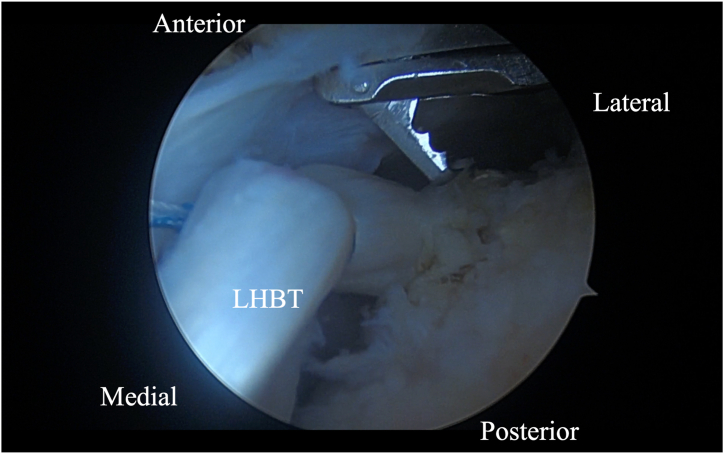
Arthroscopic image of right shoulder (lateral beach-chair position) viewed through the subacromial posterior portal showing tension being placed on the suture, pulling the biceps tendon medially, exposing more tendon length in the intra-articular space. (LHBT, long head of the biceps tendon.)

**Fig 5:**
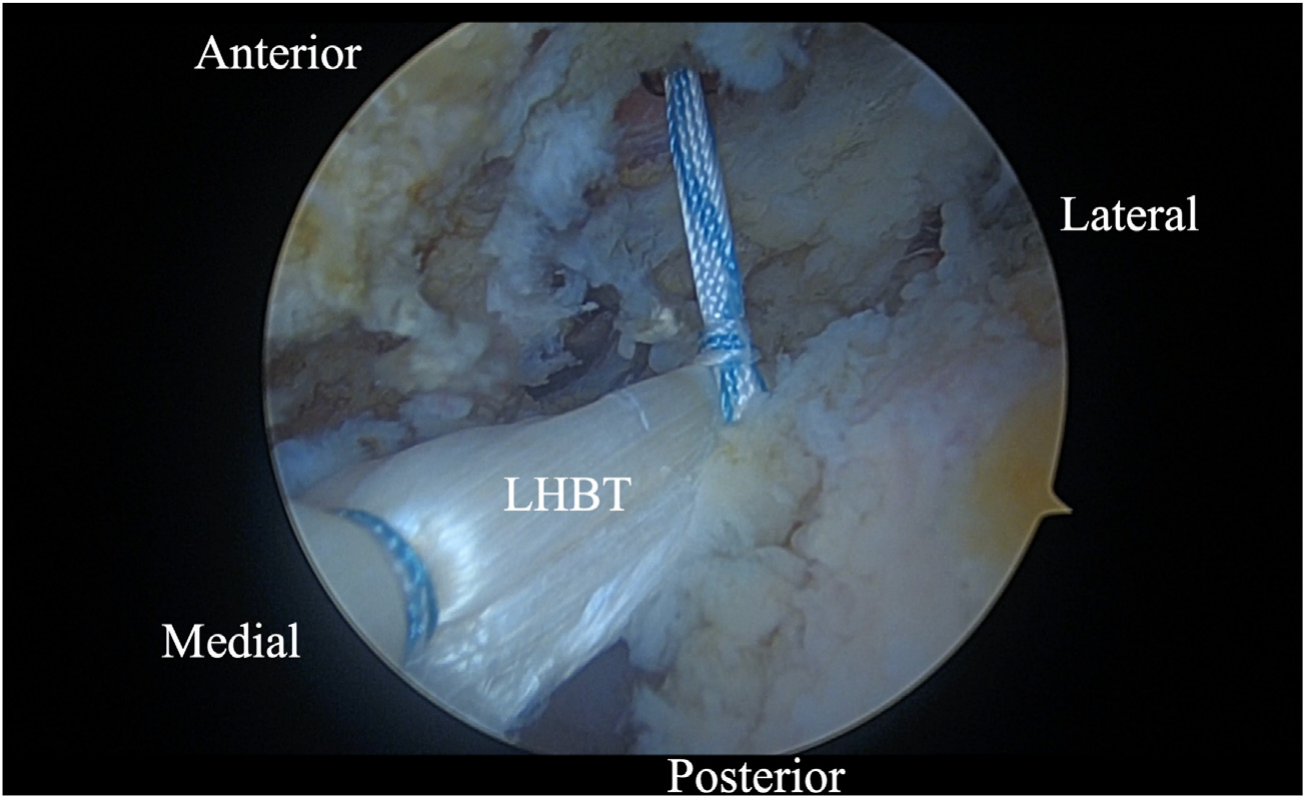
Arthroscopic image of right shoulder (lateral beach-chair position) viewed through the subacromial posterior portal showing the LHBT secured by medial and lateral luggage handle sutures in preparation for tenotomy. (LHBT, long head of the biceps tendon.)

### Tenotomy and Tenodesis

Tenotomy is performed between the 2 sutures secured on the biceps tendon, allowing for independent control of both tendon limbs (Fig 6). Biceps tenodesis is performed using the lateral biceps tendon segment. This can be performed above or below the pectoralis tendon depending on the surgeon’s preference. The author's preference is to perform this with a 2.4-mm PushLock anchor (Arthrex, Naples, FL) into the anterior aspect of the humeral head near the bicipital groove (Fig 7).

**Fig 6:**
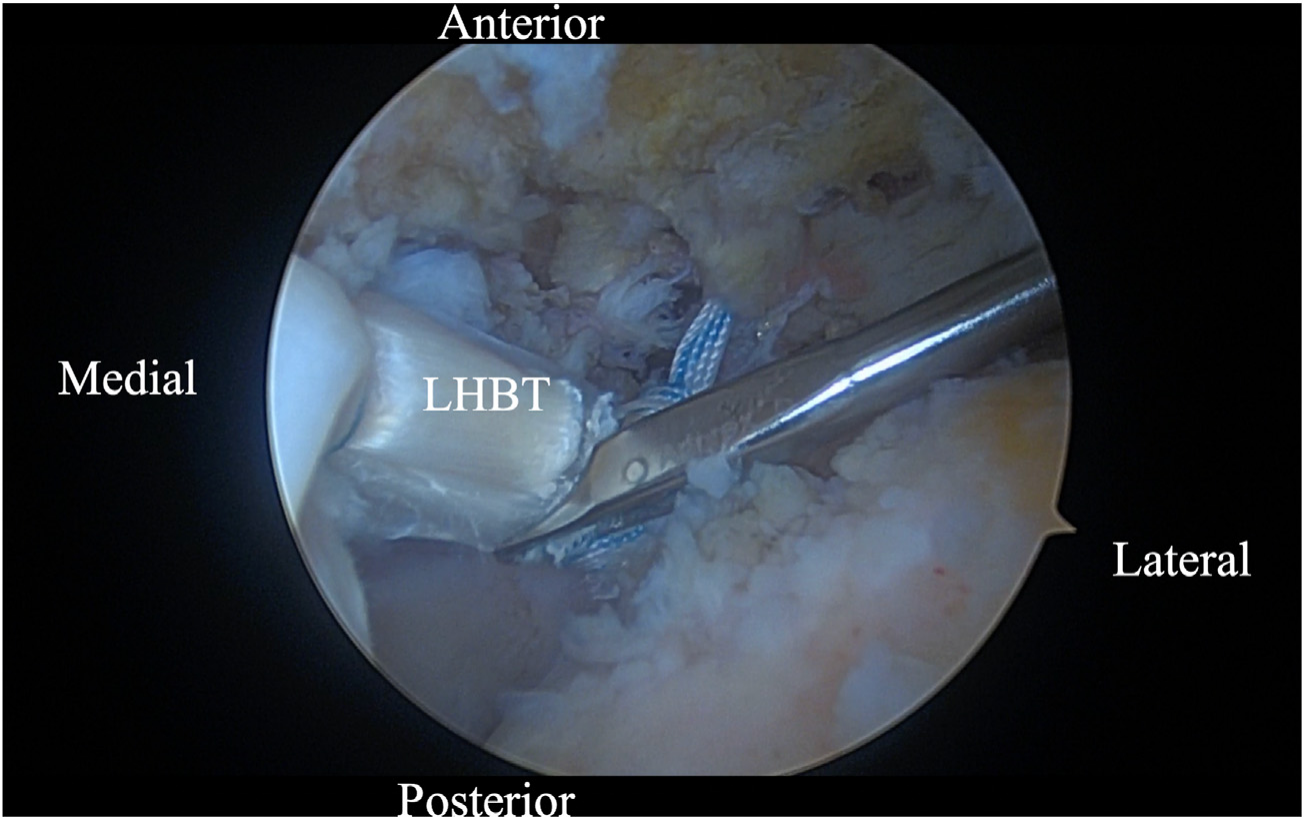
Arthroscopic image of right shoulder (lateral beach-chair position) viewed through the subacromial posterior portal showing tenotomy of the LHBT between the medial and lateral luggage handle sutures. (LHBT, long head of the biceps tendon.)

**Fig 7:**
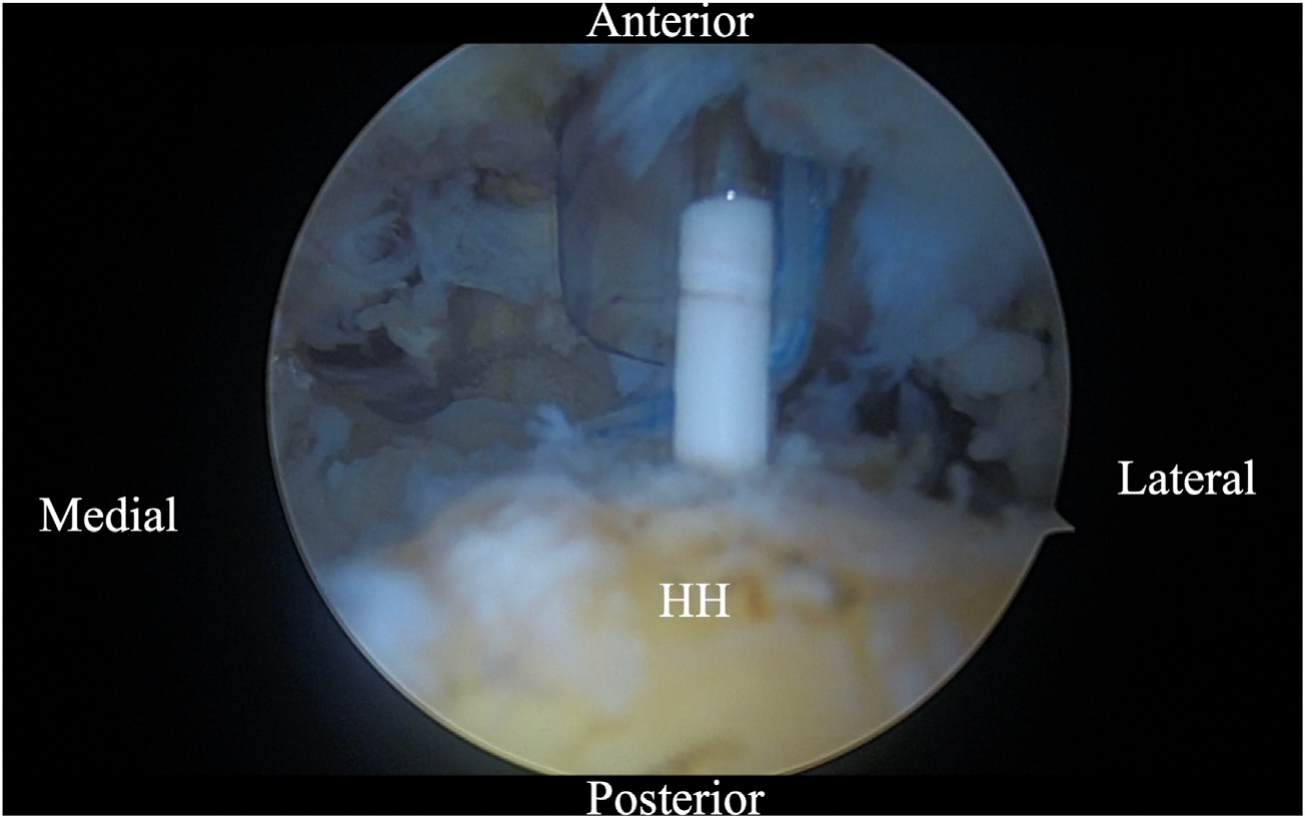
Arthroscopic image of right shoulder (lateral beach-chair position) viewed through the subacromial posterior portal showing a 2.4-mm PushLock (Arthrex) being placed in the anterior aspect of the humeral head near the bicipital groove for tenodesis of the lateral biceps tendon segment. (HH, humeral head.)

### Tendon Transfer

Attention is then focused on the more medial biceps limb that maintains its attachment to the native glenoid. The suture previously pulled medially through the Neviaser portal is extracted laterally. The biceps tendon is then manipulated with a soft-tissue grasper to sit more superiorly at the 12-o'clock position on the exposed greater tuberosity (Fig 8). Once this central superior position is obtained, it is tensioned and placed in a lateral 4.67-mm SwiveLock anchor (Arthrex) at the cartilage-greater tuberosity junction (Figs 9 and 10). Depending on the length of the tendon on examination, the surgeon can opt to use additional sutures throughout the tendon to allow it to be loaded onto more than one anchor.

**Fig 8:**
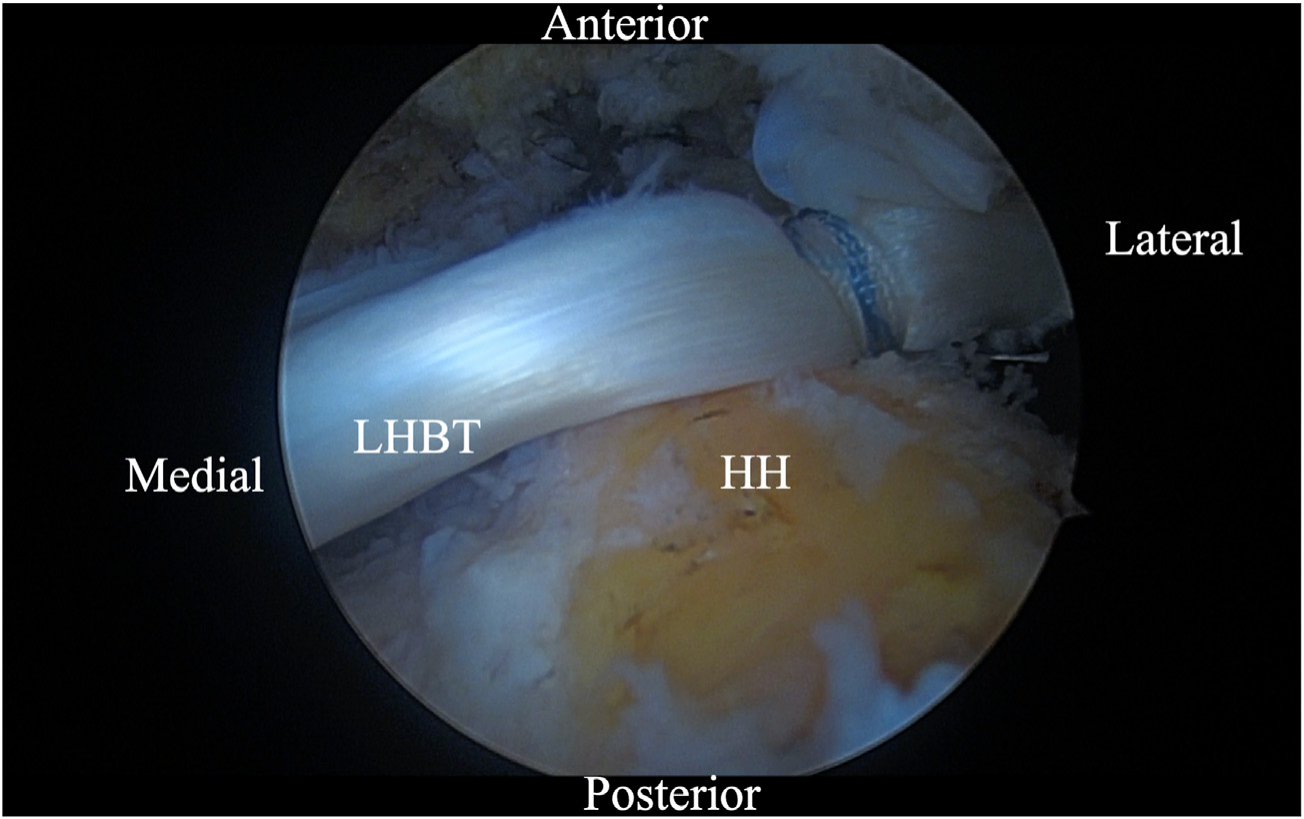
Arthroscopic image of right shoulder (lateral beach-chair position) viewed through the subacromial posterior portal showing the biceps tendon being manipulated to sit more superiorly at the 12-o'clock position on the exposed greater tuberosity. (HH, humeral head; LHBT, long head of the biceps tendon.)

**Fig 9:**
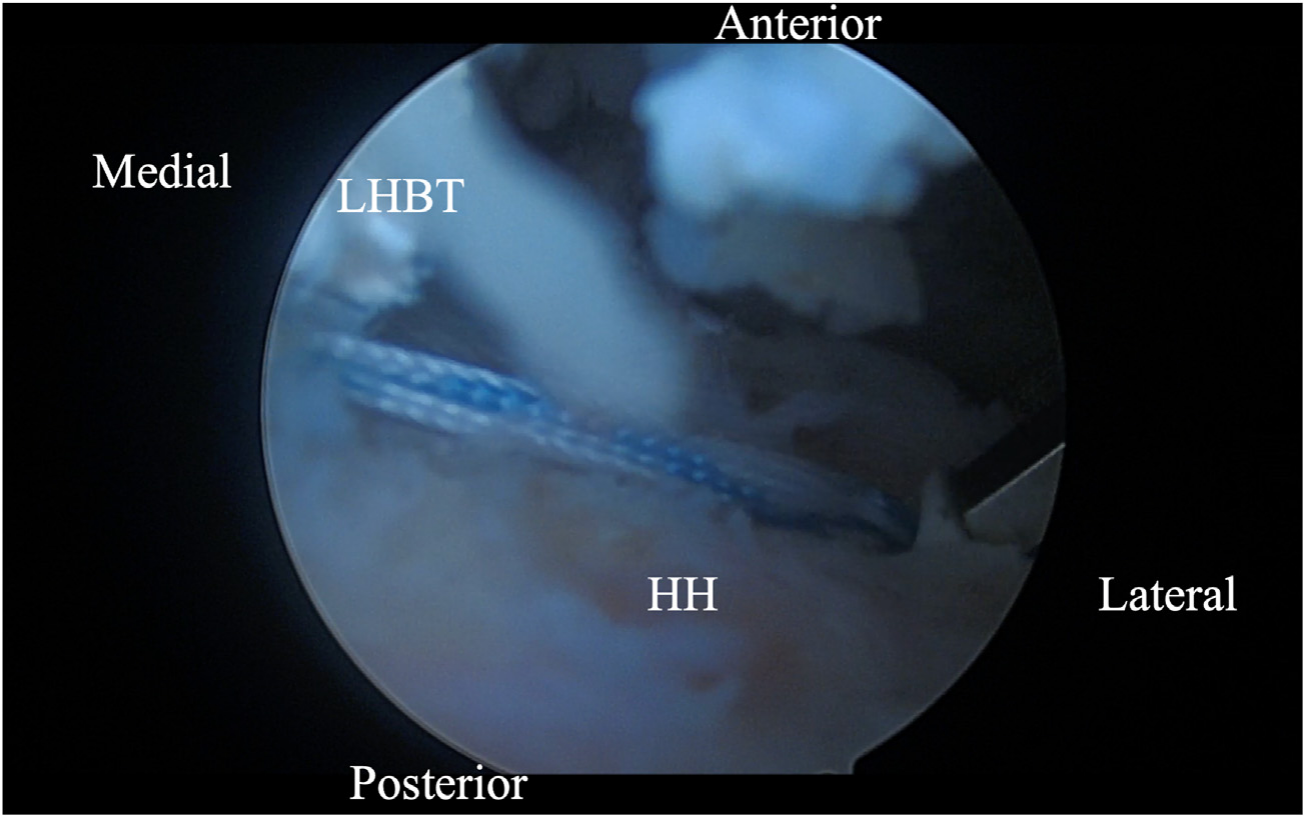
Arthroscopic image of right shoulder (lateral beach-chair position) viewed through the subacromial posterior portal showing the LHBT being tensioned laterally and placed in a 4.67-mm SwiveLock anchor (Arthrex) at the cartilage-greater tuberosity junction. (HH, humeral head; LHBT, long head of the biceps tendon.)

**Fig 10:**
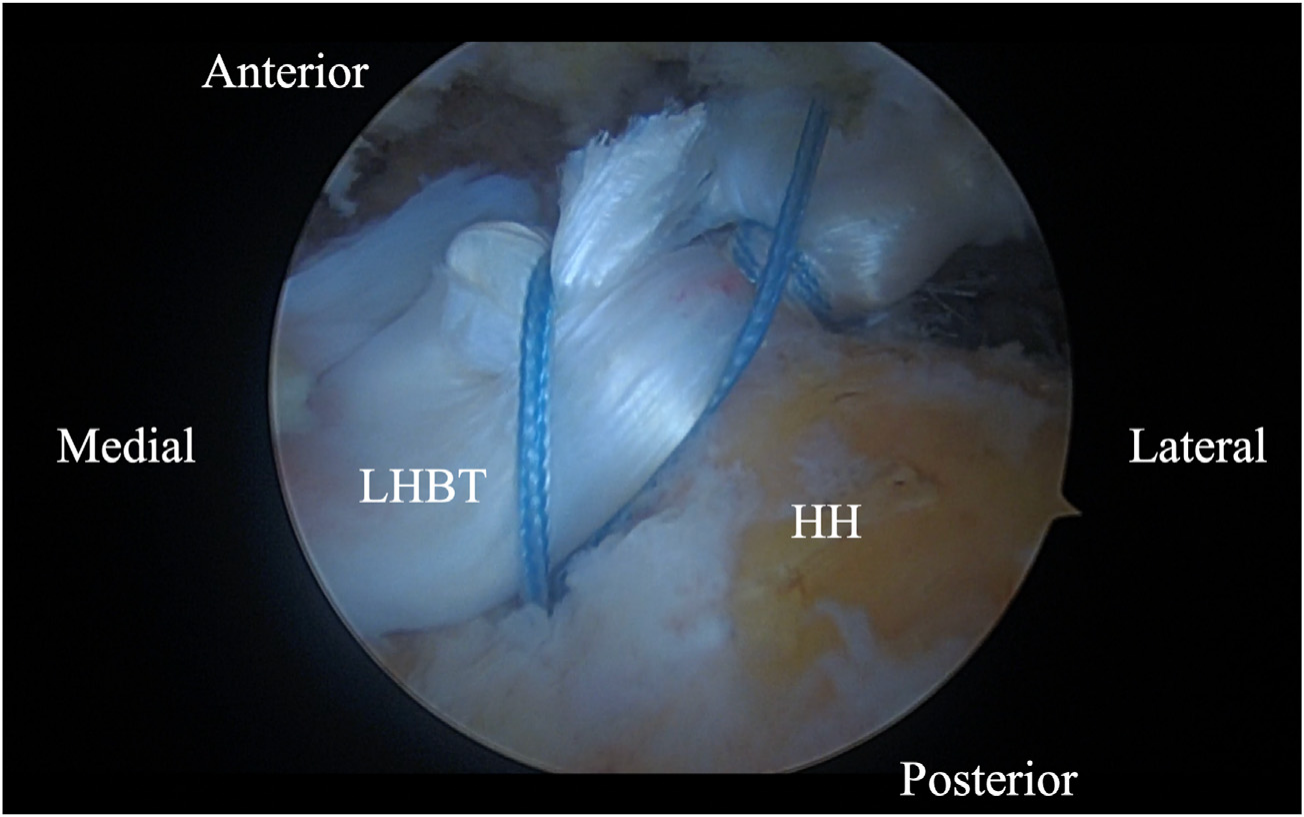
Arthroscopic image of right shoulder (lateral beach-chair position) viewed through the subacromial posterior portal showing the tensioning of the biceps augment on the greater tuberosity of the humeral head at the cartilage-greater tuberosity junction. (HH, humeral head; LHBT, long head of the biceps tendon.)

### Rotator Cuff Repair

Attention is then directed to the rotator cuff repair. This can be performed in accordance with rotator cuff appearance morphology and tissue quality. The author prefers a double-row transosseous-equivalent construct. Medially, two 2.6-mm FiberTak DR (Arthrex) anchors are placed, one anterior and one posterior to the biceps tendon transfer (Fig 11). When passed from deep to superficial, the suture from the biceps transfer anchor is passed concurrently. Laterally, two 4.75-mm knotless biocomposite SwiveLock (Arthrex) are used to compress the cuff and fixate it laterally. The sutures from the initial anchor for the biceps tendon transfer are tied in a horizontal mattress fashion, linking the rotator cuff and biceps tendon transfer constructs. Additional pathology can be treated as needed, and standard incision closure and bandage application is performed. The final BicepBrace is shown in Figure 12.

**Fig 11:**
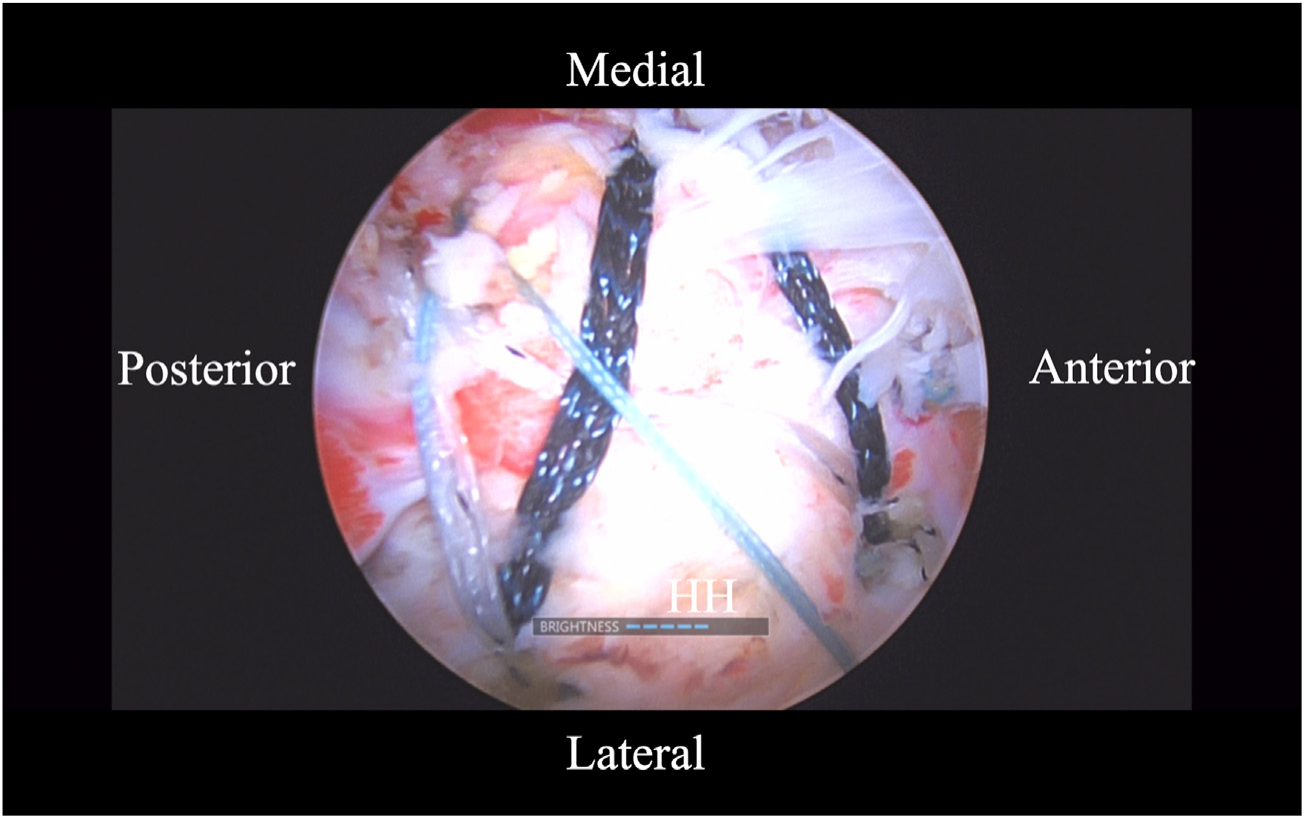
Arthroscopic image of right shoulder (lateral beach-chair position) viewed through the subacromial lateral portal showing the rotator cuff repaired in a double-row transosseous-equivalent construct. Medially, two 2.6-mm FiberTak DR (Arthrex) anchors are placed, one anterior and one posterior to the biceps tendon transfer. (HH, humeral head.)

**Fig 12:**
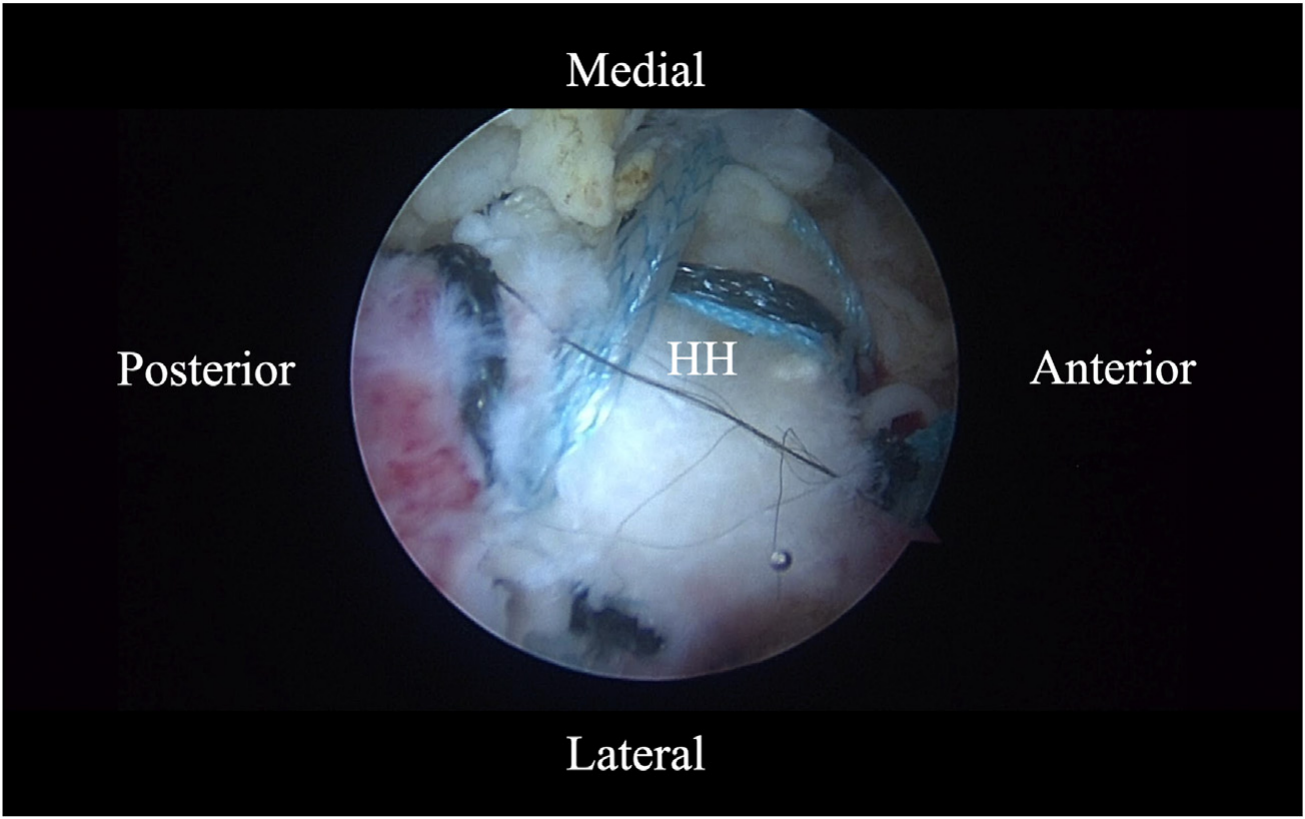
Arthroscopic image of right shoulder (lateral beach-chair position) viewed through the subacromial lateral portal the final BicepBrace construct. (HH, humeral head.)

### Rehabilitation

Rehabilitation for this technique is identical to large rotator cuff repair, consisting of immobilization and a progression of physical therapy. The rehabilitation timeline is outlined in Table 2.

**Table 2:** Postoperative Protocol

Intervention	Timeline, Weeks	Goals
Arm sling	0-6	No active or passive shoulder motion—complete immobilization
Physical therapy	6-12	Emphasis on mobility exercises
Physical therapy	12-24	Graded increase in strengthening exercises

## Discussion

Massive rotator cuff tear can be a disabling condition that lacks a definitive surgical solution. The multitude of surgical techniques have been compared with nonsurgical options such as activity modification, corticosteroid injections, and strengthening of the deltoid and periscapular musculature.^5^ Although these strategies seem to be effective treatments for nonmassive rotator cuff tears, their success rates are poor when employed for massive rotator cuff repair, with failure rates ranging from 39% to 94% reported.^42^^,^^43^

Autograft biceps tendon transfer describes a minimally invasive technique to augment rotator cuff repair or to reconstruct the superior capsule. The transferred long head of the biceps tendon acts as a stabilizer against humeral head migration and increases the structural integrity of the superior capsule, decreasing the load on the repaired massively torn tendon(s).^29^^,^^44^ In addition to increasing stabilization, it has previously been stated that biceps tendon use promotes healing and increases the likelihood of better outcomes.^45^^,^^46^ Biologic augments enhance tissue regeneration and remodeling,^46^ and the biceps tendon autograft eliminates the possibility of immunologic rejection. In addition, this technique minimizes infection risk with no extra incisions needing to be made. Instead of traditional allograft superior capsule reconstruction, healing for biceps tendon autograft requires healing on only one location (instead of 2), offering less opportunity for SCR failure. Lastly, the benefits of tuberoplasty can also be obtained if sufficient graft is harvested and secured laterally on the greater tuberosity.

The use of the long head of the biceps tendon for augmentation in the setting of rotator cuff and shoulder pathology is not unique. Other techniques have been published previously using the biceps for decreasing the width of tendon retraction in massive rotator cuff tears, SCR, and augmenting anterior cable reconstruction (Table 1). Our technique uses the tendon to directly supply a restricting force on humeral head migration with two points of fixation in order to reduce stress on the rotator cuff repair and promote healing.

Additional advantages of this technique reach beyond clinical differences. There is no added cost that is associated with other allografts. In this way, the “BicepBrace” technique can be used in situations in which cost reduction is rather important, like surgery centers. In addition, cadaveric options for grafts may not always be preferable or even possible. In countries or in situations in which cadaveric options are not available or are undesired, this alternative is completely composed of the patient's own tissue. Lastly, in situations in which tuberoplasty is desirable, the biceps tendon can be harvested, extended laterally, and can serve as an option for a potential tuberoplasty.

The potential risks associated with this procedure are similar to those of a standard massive rotator cuff repair. However, a unique risk to this approach is the increased postoperative pain that may arise from the biceps tenotomy. In addition, this technique is limited by the patient's own bone and tendon quality. The complexity of a procedure involving both tenotomy and tenodesis can result in suboptimal outcomes if not executed correctly. Specifically, the patient must have a biceps tendon that is both sufficiently long and of high quality to create 2 fully independent segments after tenotomy. Without adequate tendon length and quality, this technique cannot be effectively applied. On top of this, loosely anchored tendons (due to poor bone quality), undebrided tendon substance (whether due to tendon quality or surgeon error), and flimsy tendon sutures can all lead to poor patient outcomes such as pain and limited range of motion. The advantages and disadvantages of this technique are outlined in Table 3.

**Table 3:** Advantages and Disadvantages of the BicepBrace Technique

Advantages	Disadvantages
Free of charge—surgery center-friendly	Relies on patients having adequate bone and tendon quality
Noncadaveric option—preferable for certain circumstances	Early failure could lead to stress on rotator cuff repair
No preparation needed	
Tuberoplasty possible	
Biceps tenodesis possible	

## Disclosures

The authors declare the following financial interests/personal relationships which may be considered as potential competing interests: E.A. reports consulting or advisory with Arthrex. All other authors (T.J., G.P., B.C.) declare that they have no known competing financial interests or personal relationships that could have appeared to influence the work reported in this paper.

## Supplementary Data

Video 1 The video begins with an introductory slide, a disclosures slide, and a list of advantages for the technique. A patient prepared for surgery on their right shoulder is seated in the lateral beach chair position and a suture passer is used to denote the Neviaser portal. A slide is shown detailing indications for the procedure. The following portions are intra-operative arthroscopic video through a subacromial lateral portal. First, the distal biceps is released in the subacromial space to optimize the working length of bicep. Then, tagging of the medial and lateral biceps tendon with 2 luggage handle ties using a self-grasping suture passer is shown. Next, a tenotomy is made between the two luggage handle suture ties with arthroscopic scissors. Tenodesis is performed using a 2.4-mm PushLock anchor (Arthrex) into the anterior aspect of the humeral head near the bicipital groove. After this, there is extraction of the remaining medial luggage handle suture laterally. The BicepBrace is tensioned with SwiveLock (Arthrex). Then, medial BicepBrace fixation is shown with 2.6-mm knotless FiberTak (Arthrex). The biceps augment is now complete. Photos are shown of the complete augment in combination with a double row transosseous-equivalent construct rotator cuff repair. The video concludes with slides of rehabilitation protocol and conclusions.
